# Low levels of nestmate discrimination despite high genetic differentiation in the invasive pharaoh ant

**DOI:** 10.1186/1742-9994-7-20

**Published:** 2010-06-30

**Authors:** Anna M Schmidt, Patrizia d'Ettorre, Jes S Pedersen

**Affiliations:** 1 Centre for Social Evolution, Department of Biology, University of Copenhagen, Universitetsparken 15, DK-2100 Copenhagen, Denmark; 2 Laboratoire d'Ethologie Expérimentale et Comparée (LEEC) University of Paris 13, 99 av. J.B. Clément, F-93430 Villetaneuse, France

## Abstract

**Background:**

Ants typically distinguish nestmates from non-nestmates based on the perception of colony-specific chemicals, particularly cuticular hydrocarbons present on the surface of the ants' exoskeleton. These recognition cues are believed to play an important role in the formation of vast so-called supercolonies that have been described for some invasive ant species, but general conclusions about the role of these cues are hampered by only few species being studied. Here we use data on cuticular hydrocarbons, aggression and microsatellite genetic markers to investigate the interdependence of chemical recognition cues, genetic distance and nestmate discrimination in the pharaoh ant (*Monomorium pharaonis*), a widespread pest species, and ask whether introduced populations of this species are genetically differentiated and exhibit intraspecific aggression.

**Results:**

Microsatellite analyses of a total of 35 colonies from four continents revealed extremely high levels of genetic differentiation between almost all colonies (*F*_ST _= 0.751 ± 0.006 SE) and very low within-colony diversity. This implies that at least 34 and likely hundreds more independent lineages of this ant have spread worldwide. Aggression tests involving workers from 14 different colonies showed only low levels of aggression, even between colonies that were geographically and/or genetically very distant. Chemical analyses of groups of worker ants showed that all colonies had the same cuticular compounds, which varied only quantitatively among colonies. There was a positive correlation between geographical and genetic distance, but no other significant relationships were detected between aggression, chemical profile, genetic distance and geographical distance.

**Conclusions:**

The pharaoh ant has a global invasion history of numerous independent introductions resulting in genetically highly differentiated colonies typically displaying surprisingly low levels of intraspecific aggression, a behaviour that may have evolved in the native range or by lineage selection in the introduced range.

## Background

Unicoloniality, in which individuals from geographically separated nests intermix without aggression, has long been considered a key characteristic of invasive ants [e.g. [1-3]]. Colonies of invasive ant species can become dominant in the invaded habitats and are often termed "supercolonies" due to their sizes and huge potential for growth. Unicolonial species are assumed to have a competitive edge over other ant species, as lack of intraspecific aggression may enable a more effective resource allocation [4-7]. The precise mechanisms enabling the formation of supercolonies are a topic of some discussion [3,8]. However, several hypotheses stress the potentially important role of diversity of recognition cues and discrimination behaviour, although the hypotheses differ with respect to the role of ecological conditions and whether important traits for supercolony formation developed before or after the ants were introduced outside their native range and became invasive pests [9-11].

Cuticular hydrocarbons (CHCs) play a central role in ant communication and nestmate discrimination [12,13]. These lipids lower the risk of desiccation, can reflect environmental as well as genetic variation [13,14], and likely have evolved to function as recognisable labels enabling discrimination of nestmates from non-nestmates. Several studies have established a role for CHCs in intraspecific discrimination in ants [e.g. [15-17]], and differences in cuticular hydrocarbon profiles can often be correlated with aggression probabilities, as antagonism is expected to be expressed when odour dissimilarity exceeds a given threshold [12,18-20].

The pharaoh ant (*Monomorium pharaonis*) is a ubiquitous invasive ant species [21]. The species is mainly found in buildings and as such a common household pest. Like other invasive ant species it has a high potential for spread [2,22,23] and is polygynous, i.e. several queens reproduce in a colony. Polygyny is hypothesized to lead to lower levels of nestmate recognition relative to monogynous (single queen) species, perhaps because of the expected higher levels of genetic diversity within a colony containing multiple reproducing individuals [24]. Low levels of intraspecific aggression, if aggression depends on nestmate recognition, may therefore have facilitated the spread of pharaoh ants [2,21,25].

Here we test the hypothesis that pharaoh ants, like several other well-known invasive ant species, exhibit low levels of intraspecific aggression, and we investigate the possible role of cuticular hydrocarbons in discrimination behaviour by measuring aggression within and between laboratory strains and free-living colonies to (1) assess whether discrimination behaviour is common and (2) test whether it is primarily genetically or environmentally determined. In order to do so, we examined the extent to which the discrimination behaviour is correlated with the cuticular hydrocarbon profiles of the ants and the level of genetic differentiation between them. Finally, we studied discrimination abilities of *M. pharaonis *towards another tramp ant species, *M. destructor*, to (1) investigate whether aggression against other ant species occurred and (2) compare the chemical profiles of the two species to obtain chemotaxonomic evidence for the species identifications, (cuticular hydrocarbons are usually species specific [see e.g. [13,14]]).

We found very high levels of genetic differentiation between the different colonies and discrimination behaviour to be uncommon at first, although aggression increased with time spent in the laboratory. We found no clear correlates of inter-colonial aggression. Interspecific aggression was very high, and the two species studied had clearly distinct chemical profiles.

## Methods

### Study material

A total of 35 colony samples of *M. pharaonis *were obtained in 2004; 14 of these were live samples, either from laboratory stocks (*n *= 6), or from field collection (primarily in or on buildings, *n *= 8; Table 1). Sampling was opportunistic to represent as many different localities around the world as possible. The most extensive sampling was performed in Ghana in October 2004 to obtain colonies or colony samples from the tropics, where the species is generally more common [26]. All laboratory colonies had been reared in captivity for several years. Three samples of *M. destructor *were also collected in the field in Ghana in October 2004 (at Cape coast, Nsawam and Winniba) to be used for interspecific behavioural experiments and chemical analyses. *M. destructor *is a widespread tramp ant species, very similar to *M. pharaonis *in habitat usage as well as social structure, and the two species would typically be found at the same type of localities although not co-occurring [[27], AMS personal observation].

**Table 1:** Samples of *Monomorium pharaonis*.

Colony ID	Locality	Sample type	*P*	*k'*	***H***_**exp**_	*M*	Intraspecific tests 2004
**Gh1**	Legon, Ghana^1^	Field	1	1.45	0.141	--	52 (47; 5)
**Gh4**	Fete, Ghana^1^	Field	3	1.74	0.227	0.35*	55 (53; 5)
**Gh6**	Asuansi, Ghana	Field	3	1.64	0.199	0.52*	
**Gh7**	Biriwa, Ghana^1^	Field	3	2.27	0.390	0.44*	37 (31; 2)
**Gh8**	Koforidua, Ghana^1^	Field	3	2.64	0.374	0.47*	47 (44; 5)
**Gh9**	CRIG, Ghana^1^	Field	2	1.34	0.144	0.75	46 (42; 5)
**Gh10**	Legon, Ghana^1^	Field	2	1.27	0.073	1	45 (39; 5)
**Gh11**	Iturie, Ghana^1^	Field	0	1	0	--	60 (59; 5)
**Gh12**	CRIG, Ghana	Field	1	1.27	0.072	1	
**Gh13**	Pokwasi, Ghana	Field	3	2.01	0.362	0.70	
**Gh14**	Aburi, Ghana	Field	1	1.15	0.032	0.40*	
**Gh15**	CRIG, Ghana	Field	3	1.59	0.246	0.76	
**Gh16**	Tafo, Ghana	Field	3	2.14	0.315	0.51*	
**Gh17**	Tafo, Ghana	Field	2	1.81	0.248	0.31*	
**T**	Tingbjerg, Denmark	Field	0	1	0	--	
**I4**	Copenhagen, Denmark^1^	Field	2	1.27	0.073	0.67	48 (47; 5)
**D**	Bonn, Germany^1^	Field	1	1.05	0.008	1	51 (49; 5)
**Z**	Zürich, Switzerland	Field	1	1.19	0.046	0.67	
**TW**	Taiwan	Laboratory	1	1.25	0.124	0.03*	
**C**	Cameroon	Field	3	2.12	0.404	0.33*	
**U1**	Florida, USA^1^	Laboratory	1	1.05	0.008	0.29*	51 (51; 5)
**U2**	Texas, USA^1^	Laboratory	3	1.94	0.356	0.46*	50 (47; 5)
**U3**	Florida, USA^1^	Laboratory	3	2.03	0.318	0.51*	50 (49; 5)
**U4**	Penang, Malaysia^1^	Laboratory	1	1.05	0.008	1	61 (57; 4)
**U5**	London, UK^1^	Laboratory	3	1.74	0.332	0.66	51 (48; 5)
**U6**	Debrecen, Hungary	Laboratory	0	1	0	--	
**U7**	Warsaw, Poland	Laboratory	1	1.25	0.122	0.33*	
**U8**	Okinawa, Japan	Laboratory	2	1.35	0.144	0.33*	
**U9**	Kyoto, Japan	Laboratory	2	1.28	0.079	1	
**U10**	Moscow, Russia	Field	1	1.05	0.008	1	
**U11**	Texas, USA	Field	2	1.49	0.201	0.83	
**U17**	Lambto, Ivory Coast	Field	2	1.54	0.211	0.83	
**U18**	BCI, Panama	Field	1	1.32	0.076	0.50	
**U19**	Gamboa, Panama	Field	0	1	0	--	
**U20**	Montreal, Canada	Field	0	1	0	--	

The colony ID is the abbreviations used in further analyses and figures. The total number of polymorphic loci was four; *P *is the number of polymorphic loci for a given colony. *k' *is the average allelic richness adjusted to sample size of six. *H*_exp _is the expected average heterozygosity for each colony. * indicate statistically significant *M *values (*P *< 0.05; see Methods for details). The colony samples consisted of either workers in alcohol, live workers only, or colonies of varying sizes containing queen(-s) and brood in addition to workers. 'Field' samples were collected in the field October 2004 and 'Laboratory' samples had been kept in other laboratories as well as in Copenhagen. The number of intraspecific tests is the total number of intraspecific, inter-colonial tests conducted involving each of the populations in 2004. The number of inter-colonial tests where interactions occurred are given first in the parentheses, the number of within-colony control tests are given second in the parentheses. In 2005 colonies U1, U2, U3, U4 and Gh4 were used for behavioural tests and chemical analyses, 6 tests were made for each possible intraspecific combination of colonies as well as for the within-colony controls.^1^indicate sample used for correlation analyses including behavioural experiments and chemical analyses.

Worker samples for genetic studies were stored in 96% EtOH until analysed. Live colonies (including queens and brood) and colony samples consisting of workers to be used for behavioural and chemical studies were kept in the laboratory under uniform conditions of 25°C, 12/12 hour light/dark cycle and provided with water *ad libitum *and a diet of boiled egg yolk, cooked liver, honey, almonds and dead insects twice a week. Five viable colonies were kept for more than one year so that two independent series of behavioural and chemical tests (2004 and 2005) could be done. Since the workers' lifespan is 9-10 weeks on average [28] and unlikely to exceed four months (AMS, personal observation), and even the queens have rarely been observed to live for more than one year, repeating experiments after one year ensured that individuals tested in the second round would likely not carry any cues derived from their previous environments. Of the five colonies tested again in 2005, four were originally laboratory colonies and one was a field colony, collected in Ghana in 2004 (cf. Table 1).

### Genetic analyses

The genetic variation and differentiation of the colonies was examined based on four microsatellite loci for which primers were developed for *M. pharaonis *(Table 2). DNA was extracted from 30 workers from each colony (except 6 workers from U19) by each specimen being crushed in 200 μl 5% Chelex solution. The primers were run in separate PCR reactions in 20 μl reaction volumes with 1 μl extract as template, reaction buffer, 25 mM MgCl_2_, 0.5 mM GATC, 10 μM forward and reverse primers and 0.1 μl Taq gold polymerase. The PCR reaction conditions consisted of an initial denaturing step of 95°C for 10 min, followed by 27 cycles of 95°C for 30 s, 60°C for 30 s, and 72°C for 30 s, and finally an extension step at 72°C for 1 h. The PCR products were run on 2% ethidium bromide agarose gels, and subsequently run on 5% polyacrylamide gels using an ABI PRISM^® ^377 DNA automated sequencer with Rox500 as internal standard. The four loci can be multiplexed on the ABI. GENESCAN 3.1 and GENOTYPER 3.1 were used to score the alleles.

**Table 2:** Microsatellite loci developed and analysed in this study

Locus	Primer sequences (5'-3')	Repeat motif	GenBank accession no.	No. of alleles	Size range (bp)
*Mp1*	f: GCCAATGGTTTAATCCCTCA	(AG)_3_AA(AG)_23_	HM587312	15	192-279
	r: TCATACTGCGTGTGCCTTTC				
*Mp3*	f: ACAAGGTAAGTCGCCACCAT	(GT)_20_	HM587313	9	127-147
	r: TCGTGATAATTCGCGATGAA				
*Mp12*	f: TGGCCAAAAGTATCCAGGAG	(AC)_8_	HM587314	2	130-132
	r: TCGTCGAAAGTATCGAAGTAAAC				
*Mp13*	f: CCCATTGAGATTGCGGCAT	(AC)_10_(GC)_8_ACA(AC)_10_	HM587315	9	281-298
	r: GCACAGGCACGTAACGATT				

Data on allele number and size range are for the total sample of 35 *Monomorium pharaonis *colonies analysed (*n *= 1026 individuals).

### Cuticular hydrocarbon analyses

Groups of 5 workers per colony were frozen at -20°C after which surface chemicals were extracted by washing in 20 μl pentane for 10 min. Three μl of this solution was injected into an Agilent Technologies 6890N Gas Chromatograph (GC) equipped with a capillary column (HP5MS 30 m × 250 μl × 0.25 μm). The injector was a split-splitless type with helium carrying gas at 1 ml/min. The initial temperature was 70°C, and was increased at 20°C/min to 280°C, then to 310°C at 2°C/min, and held for 5 min. The GC was coupled with a 5375 Agilent Technologies Mass Spectrometer, using 70 eV electron impact ionization. Compounds were identified on the basis of their mass spectra, and comparison with standards and published spectra. One sample was run for each colony. A single, concentrated reference of 25 pooled individuals (colony U1 used) was analysed to ascertain the identity of the peaks used in the subsequent analyses.

### Behavioural experiments

Standardized aggression tests were performed where dyadic encounters were continuously observed over 10 min following Giraud et al. [4] with the modification that we allowed the ants to habituate separately for 1 min within the arena before the start of the observation period. All interactions were scored in easily distinguishable categories on a scale from 0 to 5: levels 0 (no response) and 1 (antennation) were considered neutral, non-aggressive behaviours, and levels 2 (escape), 3 (gaster raising), 4 (biting), and 5 (fighting, which involved prolonged biting, often ensuing in one ant biting onto the other for the remainder of the observation period) were considered antagonistic. Within 6 weeks from field collection in 2004 a mean of 3.9 tests (median = 5, range 1-6) were carried out for each intraspecific *M. pharaonis *colony pair as well as for the controls (i.e. within-colony controls). 5 tests were conducted for each interspecific combination of *M. pharaonis *vs. *M. destructor *(except Gh7 with one test in two of the combinations) and for the *M. destructor *within-colony control tests, to obtain a matrix of 635 tests in total. In 2005 a sub-sample of 5 of the *M. pharaonis *colonies was tested, with 6 tests for each possible intraspecific combination of colonies as well as for the within-colony controls, resulting in 90 intraspecific tests in total. The tests were not done blind as regards the colony origin of the ants, but the interaction categories were clearly distinguishable, thus preventing any subjectivity in the interpretation of the behaviour. Moreover, the experimenter had no prior knowledge of the genetics or chemical profiles of the colonies. No ants were used for more than one test to avoid any possible effects of familiarization.

### Statistical analyses

The genetic differentiation (*F*_ST_) between the colonies as well as measures of allelic richness (*k'*) and inbreeding at the colony level (*F*_IS_) were calculated using FSTAT 2.9.3.2 [29] with significance testing based on 15,000 randomisations. Standard errors were estimated by jack-knifing over colonies. As the value of *F*_ST _is affected by the allelic diversity at the marker loci applied, we further calculated the standardised *F'*_ST _[30] and the estimator *D*_est _[31] as alternative quantifications of the genetic differentiation, making comparisons with studies based on other marker loci possible [32].

The program M_ratio [33], was applied to test for signs of population bottlenecks as *M *is the ratio of allele number to range in allele sizes (*M *= *k*/*r *), and a significant reduction in *M *relative to the expected distribution is interpreted as an indication of a recent reduction in population size. The estimates of *M *were based on simulation of 10,000 replicates for the polymorphic loci. We applied the non-spatial genetic mixture analysis implemented in BAPS 5.3 [34] to cluster colonies that are likely to be genetically similar e.g. from having a common population history or representing the same supercolony [35,36]. The maximum number of genetically divergent groups (*K*) was set to the number of colonies included in the data set, and each analysis was repeated 10 times to ensure consistency of results between different runs. The genetically divergent groups of colonies identified this way are interpreted as colony lineages. Based on the observed number of lineages in our sample we estimated the total number of such lineages in the world by the Chao1 estimator of Chao [37] using the program SPADE [38]. Furthermore, a principal component analysis (PCA) of the microsatellite allele frequency data was performed in PCAGEN 1.3.1 [39], as an exploratory technique to visualise colony or population associations possibly reflecting the phylogeographic history of the species.

For the data on chemical profiles Statistica 7 (StatSoft) was used to perform PCA on peak areas, after log-ratio transformation according to Aitchison [40]. In addition, principal components combined explaining a minimum of 95% of the variation were found in JMP.IN 5.1 and subsequently used for calculating the inter-sample chemical (Euclidian) distances as a measure of their relative differences. Cluster analyses were performed based on the cuticular hydrocarbon data, see additional file 1.

Mantel tests for possible correlations between the chemical and behavioural, genetic as well as geographical distances were performed in FSTAT [29], all based on 10,000 permutations. The results of the subset of colonies that were studied in both 2004 and 2005 were compared with MANOVA. The behavioural data were analysed using Kruskal-Wallis test with Dunn's post-hoc test and Mann-Whitney *U*-test for the 2004 and 2005 data respectively; Fisher's exact test was applied to compare the overall occurrence of aggression between the two years.

## Results

### Genetic diversity and differentiation

Table 2 gives information on the four microsatellite markers that were developed for this study. At one locus (*Mp12*) only two alleles were found, and all colonies except one (TW) were monomorphic for one of these alleles, while TW was monomorphic for the other. The genetic diversity within colonies was generally very low, as over 2/3 of the colonies only had two polymorphic loci, and five of these colonies were monomorphic at all four loci (Table 1). The allelic richness (*k'*) was 1.47 ± 0.07 SE, the expected heterozygosity (*H*_exp_) was 0.153 ± 0.023 SE on average across colonies and, as expected, these measures of the genetic diversity were highly correlated (Spearman *r *= 0.977; *P *< 0.0001). The calculations from the M_ratio program indicated that 14 of the colonies had likely gone through a reduction in the effective number of reproducing individuals (Table 1).

**Figure 1:**
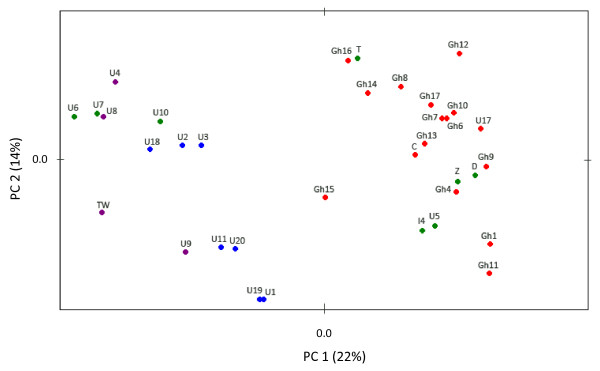
**Principal Component Analysis of microsatellite allele frequencies of *Monomorium pharaonis *colonies**. Colour coding is by continent; red: Africa, green: Europe, blue: America, purple: Asia. Colony labels are as in Table 1. The explained variances are given in parentheses.

There were no signs of deviation from random mating within the colony samples (*F*_IS _= -0.021 ± 0.005 SE; *P *= 0.82), confirming that each colony formed its own breeding population as seen in other invasive ant species (e.g. [35]). We consistently found very high and statistically significant values of interpopulation genetic differentiation (mean *F*_ST _= 0.751 ± 0.006 SE; *P *< 0.0001; corresponding to *F'*_ST _= 0.891 and *D*_est _= 0.561; see also additional file 2 for estimates of pairwise *F*_ST_). Only two colonies (U1 from Florida and U19 from Panama) were grouped as genetically similar by the BAPS analysis, as also reflected in the PCA plot (Fig. 1), and this result (*K *= 34) had strong support by a posterior probability *P *= 1.000 in all 10 runs. Consequently, the group of these two colonies and the 33 remaining colonies were all significantly genetically divergent, indicating that there were 34 independent lineages represented in our sample. Extrapolating this finding using the Chao estimator implies that the total number of such lineages of pharaoh ants in the world should be counted in hundreds, maybe even thousands (*S*_Chao1 _= 579; 140-3005 95% C.I.).

### Cuticular hydrocarbon analyses

**Figure 2:**
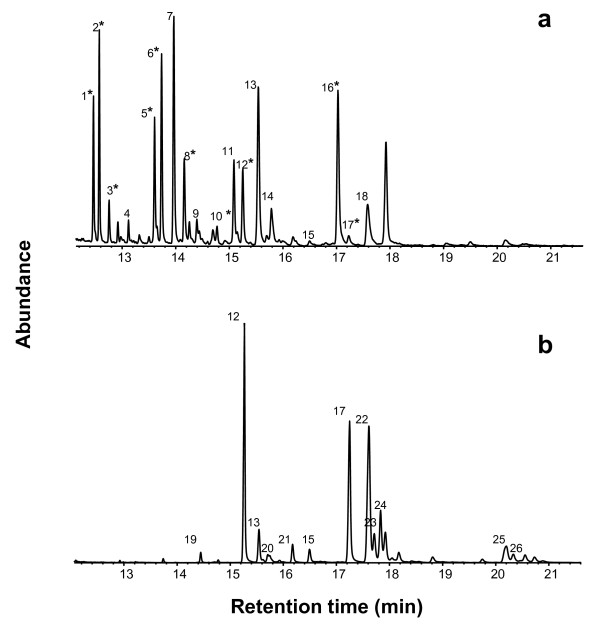
**Cuticular hydrocarbon profiles of *Monomorium pharaonis *(a) and *M. destructor *(b)**. The numbers on the peaks mark the identified hydrocarbons: (1*) C25:1; (2*) C25; (3*) 9-,11-,13-MeC25; (4) C26; (5*) C27:1; (6*) C27; (7) 9-,11-,13-MeC27; (8*) 11,15-diMeC27; (9) 3-MeC27; (10*) 3,11-diMeC27; (11) C29:1; (12*) C29; (13) 11-,13-,15-MeC29; (14) 11,15-diMeC29; (15) 3,11-diMeC29; (16*) C31:1; (17*) C31; (18) 11-,13-,15-MeC31; (19) C28; (20) 5-MeC29; (21) C30; (22) 11-,13-MeC31; (23) 7-MeC31; (24) 5-MeC31; (25) 11-,13-MeC33; (26) 7-MeC33. The numbers with an asterisk denote the hydrocarbons that explain most of the variation in the overall principal component analysis, i.e. numerical value of factor loadings > 0.7. Factor loadings for these compounds were: (1) -0.736; (2) -0.835; (3) -0.867; (5) -0.745; (6) -0.885; (8) -0.768; (10) 0.797; (12) -0.751; (16) 0.902; (17) -0.841.

**Figure 3:**
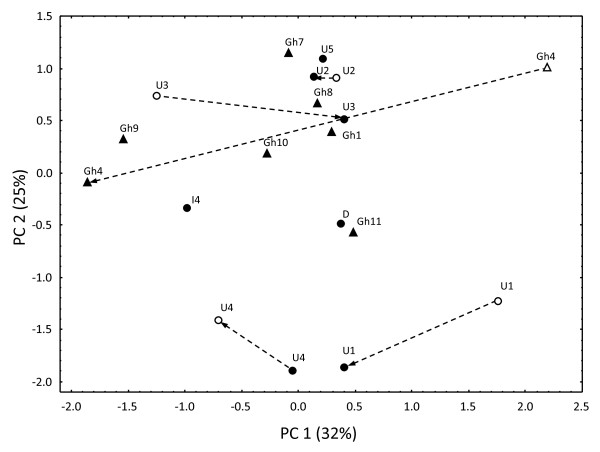
**Principal Component Analysis of cuticular hydrocarbons of *Monomorium pharaonis *colonies**. Filled and open symbols indicate colonies analysed in 2004 (*n *= 14) and 2005 (*n *= 5), respectively. Colonies from Ghana are shown by triangles, whereas the rest are shown by circles. Dashed lines are drawn between the symbols representing colonies analysed in 2004 and 2005. Colony labels are as in Table 1. The explained variances are given in parentheses.

The cuticular profile of *M. pharaonis *was characterized by 18 different hydrocarbons, which were a mix of unbranched, mono- and di-methyl-branched alkanes with 25 to 33 carbons and unsaturated hydrocarbons (Fig. 2a). As these hydrocarbons were invariably present, our analysis focused on quantitative differences among *M. pharaonis *colonies. For the 2004 and 2005 data a combined PCA extracted 5 principal components with eigenvalues larger than 1 (eigenvalues and percent variance explained by each of the 5 components: 5.83, 32%; 4.53, 25%; 2.22, 12%; 1.52, 8%; 1.10, 6%). PCA of all cuticular profiles did not provide clear groupings in either year (Fig. 3), and there were no significant differences in inter-colonial chemical distances between the years (MANOVA on first four principal components, *F*_3,2 _= 0.57; *P *= 0.68), indicating that laboratory rearing had not lead to convergence of the cuticular profiles. From Fig. 3 there seem to be changes between years, but these are not consistently in any direction, although our sample size of five colonies is too small to test this statistically or to test directly for significant colony-specific changes between years.

**Figure 4:**
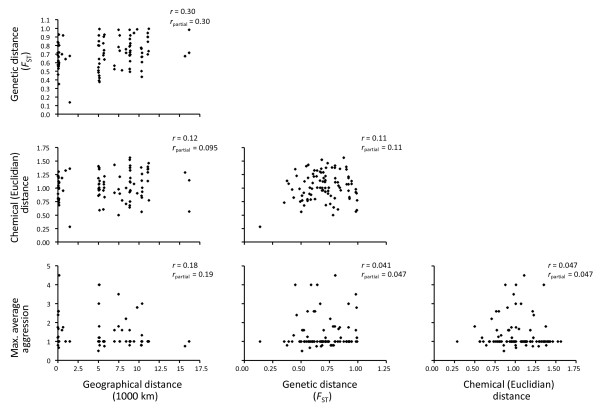
**Association of genetic, chemical, behavioural, and geographical distances between colonies of *Monomorium pharaonis***. Correlation coefficients as well as partial correlation coefficients are given for each plot. Mantel tests yielded insignificant correlations (*P *> 0.09), except for genetic vs. geographical distance (*P *= 0.005).

Ten hydrocarbons accounted for most intercolony variation (i.e. factor loadings > 0.7) and were a mix of different types, branched as well as unbranched (see legend of Fig. 2a). For the 2004 data there was no significant correlation between chemical distances and level of aggression across colonies (Mantel test, *n *= 14, *r *= 0.047; *P *= 0.65; Fig. 4). For further illustration of the seeming lack of geographical clustering based on the chemical data see Ward tree based on hierarchical cluster analysis in additional file 1.

When compared with *M. destructor*, the *M. pharaonis *chemical profiles were clearly qualitatively different: *M. destructor *consisted of 12 hydrocarbons of which only four were also found in *M. pharaonis *(Fig. 2).

### Aggression assays

In 45 behavioural tests no interactions between the ants were observed, leaving 590 tests for further analyses. Aggression occurred in 23% (21 out of 91 combinations) of the intraspecific colony pairs obtained in 2004. The average aggression level was 0.99 ± 0.05 SD for 14 within-colony control pairs (median = 1, interquartile range = 0), 1.37 ± 0.82 for 91 intraspecific pairs (median = 1, interquartile range = 0.29), and 3.68 ± 0.98 for 42 interspecific pairs (median = 3.8, interquartile range = 1.55). There was a significant overall difference (Kruskal-Wallis test, *n *= 147, *H *= 85; *P *< 0.0001), the difference between intra- and interspecific aggression also being significant (Dunn's post-hoc test, *n *= 2; both *P *< 0.001), but no significant difference was found between intraspecific and within-colony (control) aggression (Dunn's post-hoc test, *n *= 2; *P *> 0.05), reflecting a generally rare occurrence of intraspecific aggression. The level of aggression between different colonies of *M. pharaonis *did not depend on the presence of queens (Kruskal-Wallis test on queenright-queenright, queenright-queenless and queenless-queenless colony pairs, *n *= 91, *H *= 2.96; *P *= 0.23). The second series showed higher overall occurrence of intraspecific aggression after one year: whereas aggression was observed in only 1 of the 10 colony combinations in 2004, it was observed in 7 of the 10 combinations in 2005 (Fisher's exact test; *P *= 0.02) and there was a significant difference between intraspecific and within-colony (control) aggression in 2005 (Mann-Whitney *U*-test, *n *= 15, *U *= 5; *P *= 0.019).

### Correlation analyses

The Mantel tests for association of geographical, genetic, chemical, and behavioural distances between colonies (*n *= 14) only showed a significant positive correlation for the combination of genetic and geographical distance (Fig.4; *r *= 0.30, *P *= 0.005; see Table 1 for colony IDs). Tests for correlation between genetic and geographical distances were also performed for the entire sample of 35 *M. pharaonis *colonies, as well as for the sub-set from Ghana. The correlation between genetic and geographical distance was also significant for the 35 colonies (*r *= 0.25, *P *= 0.0001). This indicates that there might be genetic isolation by distance between colonies at the global scale. However, the same correlation was not found when the sub-set of colonies from Ghana was analysed separately (*n *= 14, *r *= 0.06; *P *= 0.57). Thus this apparent isolation by distance appears to be a product of generally very high levels of genetic differentiation between the colonies, combined with slightly lower levels of differentiation found among the African samples, which, due to their high representation (16 of 35 colonies), skew the distribution in the shorter distance end of the geographical scale.

## Discussion

The very high genetic differentiation between almost all colonies studied suggests that pharaoh ants have made their way to at least four different continents through numerous human-mediated introductions resulting in at least 34 and likely hundreds more independent lineages. Colonies appear to have frequently gone through genetic bottlenecks in the introduction process, resulting in possibly isolated colonies with low genetic diversity and extremely high intercolonial differentiation. In fact, to our knowledge, the *F*_ST _value found (0.751) is the largest estimate for genetic differentiation at nuclear loci reported for any non-selfing sexually-reproducing organism. The extreme genetic divergence among lineages is not a statistical artefact of analysing markers of limited allelic diversity, as also the unbiased estimators *F'*_ST _and *D*_est _are exceeded by only one similar species among the studies reviewed by Heller and Siegismund [32]. Hence, the pattern of dispersal and organisation in independent supercolonies closely resembles that of other invasive ant species like the Argentine ant (*Linepithema humile*), *F*_ST _= 0.419; [36] and the invasive garden ant (*Lasius neglectus*), *F*_ST _= 0.334; [41], but is characterised by an even larger degree of isolation, likely reflecting a longer invasion history.

We successfully identified 26 cuticular hydrocarbons from the profiles of *M. pharaonis *and *M. destructor *and found only quantitative variation between *M. pharaonis *colonies but clear qualitative differences between the two species (Fig. 2). Although there were extremely high levels of genetic differentiation between the *M. pharaonis *colonies, which led us to speculate whether several cryptic species might be involved, the cuticular data provide chemical evidence that this is not the case. The PCA of the cuticular profiles (Fig. 3) did not disclose any geographical or other groupings among the apparently chemically very similar *M. pharaonis *colonies investigated. Previous studies on the little fire ant (*Wasmannia auropunctata*) and the Argentine ant (*Li. humile*) have shown quantitative as well as qualitative differences in chemical profiles between native as well as introduced colonies, with introduced colonies generally having fewer compounds in their profiles [42,43]. These findings indicate that CHC analyses could potentially be an indirect way of discerning between ants from the native and introduced ranges. However, in our sample all pharaoh ants analysed were chemically qualitatively similar, and had relatively few compounds in their profiles relative to other ant species. This indicates that they are either all from introduced colonies, or that the pattern found in *W. auropunctata *and *Li. humile *does not apply to this species.

Recent investigations suggest that double-bonded and methylated hydrocarbons may be more informative for nestmate discrimination than other hydrocarbons and that a combination of different classes of hydrocarbons may be necessary for efficient discrimination [17,44-47]. The presence of several methylated hydrocarbons in *M. pharaonis *cuticular profiles therefore indicates that information for nestmate recognition is potentially available.

For another invasive ant species, the Argentine ant, Suarez et al. [48] found a correlation between cuticular chemistry and behaviour even after a year of laboratory rearing, suggesting that nestmate discrimination is a direct function of chemical distance. Likewise, Liang and Silverman [16] and Liang et al., [49] found strong correlational evidence for the importance of environmentally derived hydrocarbon cues in the discrimination behaviour of Argentine ants. The lack of correlation found in our study indicates that there is no similarly simple role of cuticular hydrocarbons in *M. pharaonis*. Interestingly, between-colony aggression had increased after one year of laboratory rearing, the precise mechanism of this, however, is yet to be studied. Like other invasive ants [3,6], *M. pharaonis *was less aggressive against conspecifics than would be expected based merely on the large geographical distances. For example, there is >8000 km between the completely non-aggressive colonies Gh1 and U1, which are highly genetically distinct (*F*_ST _= 0.824; Bonferroni corrected *P *< 0.05). Although queenless workers are often found to be less aggressive than queenright workers [50], this is not the case for many species including two other invasive ant species, the Argentine ant and the fire ant [51,52]), giving us good reason to assume that this would also not be the case for pharaoh ants. As our results show no difference in the average level of aggression in interactions involving queenright and/or queenless colonies, we believe that using workers from queenless colonies has not affected our results.

The absence of aggression between distantly related colonies is remarkable and may be a characteristic of the pharaoh ant as an invasive species. As has been suggested for *La. neglectus *[11] and *La. austriacus *[53], a reduced diversity of recognition cues or a general abandoning of aggression towards conspecifics can be possible pre-adaptations in the native range for the later formation of invasive supercolonies. Alternatively, selection for increased acceptance may have taken place as lineage selection among the large number of isolated colonies in the introduced area [8,54], with the likely benefit of having fewer false rejections of colony members in a changed ecological setting where different lineages will rarely compete directly [55,56]. One could question whether low levels of intraspecific aggression would provide species like the pharaoh ant with a competitive edge relative to other ant species as it seems likely that they do not necessarily face very intense interspecific competition in their introduced range. This is because they primarily - possibly exclusively - occupy habitats that are characterized by human disturbance and therefore contain few important native competitors. Studying the species' distribution patterns on a more local scale may provide useful knowledge to evaluate which selective pressures they are exposed to, and whether low levels of intraspecific aggression potentially enabling the merger of introduced colonies may be advantageous. As successful ant invaders appear well adapted to survival in disturbed environments, the role of disturbance in ant invasions [57,58] as well as in the evolution of unicoloniality [59,60] is currently debated.

Absence of detectable aggression or intraspecific discrimination behaviour as measured in tests such as those applied in this study does not necessarily imply that the ants are not able to tell nestmates from non-nestmates, only that they do not react on perceived differences by exhibiting aggression [see also e.g. [53,60]]. The exact mechanism of the low level of aggression among pharaoh ants is not clear from the present study, and may therefore not reflect a lack of information, but rather an impairment in the perception of variation, a modified acceptance threshold in the colonies tested [9,61], or a lack of response to perceived cues [53]. Although we cannot rule out that other substances, such as glandular secretions, may affect nestmate discrimination in pharaoh ants, the possibility that a neurological perception mechanism to ignore kinship information may have been convergently selected in invasive ants could provide an exciting area for further study.

## Conclusions

We found that the investigated colonies of pharaoh ants were very highly genetically differentiated, but did not exhibit correspondingly high levels of intraspecific aggression or divergence in cuticular hydrocarbon profiles. Uniform rearing in the laboratory appeared to increase the observed level of aggression, which, if supported by additional studies, could be seen as indirect evidence for genetically based nestmate discrimination behaviour that is otherwise not expressed in more diverse environments.

No colonies stood out in terms of genetic or chemical composition, and it is therefore not possible to draw any conclusions regarding the geographical origin of the species. Further research is needed to elucidate the population genetics of the species on a finer geographical scale as well as the role of low levels of aggression towards conspecifics in the species' invasive abilities. As pharaoh ants can be kept in the laboratory and colonies crossed, it lends much promise for further investigations on these questions.

## Competing interests

The authors declare that they have no competing interests.

## Authors' contributions

AMS collected the ants and conducted the field work, carried out the behavioural assays and genetic lab work and analyses, participated in the chemical analyses and the design of the study and drafted the manuscript. PdE participated in the chemical analyses. JSP participated in the design of the study. All authors revised the draft manuscript and approved the final manuscript.

## Supplementary Material

Additional file 1 **Ward trees based on hierarchical cluster analyses of CHC data (a) *Monomorium pharaonis *and (b) *M. pharaonis *and *M. destructor***. ID codes for *M. pharaonis *are like those given in Table 1, and the *M. destructor *samples have been added as Md1, Md2, and Md3. (a) was constructed in JMP.IN 5.1 based on Euclidian distance measures obtained from 18 identified cuticular hydrocarbons in *M. pharaonis *(see Methods for further details). The colours have been randomly assigned to the different branches. (b) was constructed in JMP 8.0 based on simple presence-absence data of the 26 cuticular hydrocarbons found in *M. pharaonis *and *M. destructor *combined; the *M. destructor *branches have been marked purple. Click here for file

Additional file 2 **Estimates of pairwise genetic distances (*F*_ST_) between sampled colonies of *Monomorium pharaonis *based on four microsatellite loci analysed and calculated in FSTAT **(Table 2). Click here for file
