# Supplementary material for: Low levels of nestmate discrimination despite high genetic differentiation in the invasive pharaoh ant
Source: Front Zool. 2010 Jun 30;7:20. doi: 10.1186/1742-9994-7-20 (PMC2907370; doi:10.1186/1742-9994-7-20)
Supplement: Additional file 1 — Ward trees based on hierarchical cluster analyses of CHC data (a) Monomorium pharaonis and (b) M. pharaonis and M. destructor. ID codes for M. pharaonis are like those given in Table 1, and the M. destructor samples have been added as Md1, Md2, and Md3. (a) was constructed in JMP.IN 5.1 based on Euclidian distance measures obtained from 18 identified cuticular hydrocarbons in M. pharaonis (see Methods for further details). The colours have been randomly assigned to the different branches. (b) was constructed in JMP 8.0 based on simple presence-absence data of the 26 cuticular hydrocarbons found in M. pharaonis and M. destructor combined; the M. destructor branches have been marked purple. [file 1742-9994-7-20-S1.DOC]

a

I4

Gh9

Gh4

D

Gh11

Gh7

U2

U3

Gh1

Gh8

Gh10

U5

U1

U4

b

Gh1

Gh4

Gh7

Gh8

Gh9

Gh10

Gh11

I4

D

U1

U2

U3

U4

U5

Md1

Md2

Md3
